# *In Situ* Self Assembly of Nanocomposites: Competition of Chaotic Advection and Interfacial Effects as Observed by X-Ray Diffreaction

**DOI:** 10.3390/nano5010351

**Published:** 2015-03-17

**Authors:** Dilru R. Ratnaweera, Chaitra Mahesha, David A. Zumbrunnen, Dvora Perahia

**Affiliations:** 1 Department of Chemistry, Clemson University, Clemson, SC 29634-0973, USA; E-Mail: dratnaw@clemson.edu; 2 Department of Mechanical Engineering, Clemson University, Clemson, SC 29634-0921, USA; E-Mails: cmahesh@clemson.edu (C.M.); zdavid@clemson.edu (D.A.Z.)

**Keywords:** assembly, nanocomposites, chaotic advection, nylon, smart blending, X-ray diffraction

## Abstract

The effects of chaotic advection on the *in situ* assembly of a hierarchal nanocomposite of Poly Amide 6, (nylon 6 or PA6) and platelet shape nanoparticles (NPs) were studied. The assemblies were formed by chaotic advection, where melts of pristine PA6 and a mixture of PA6 with NPs were segregated into discrete layers and extruded into film in a continuous process. The process assembles the nanocomposite into alternating pristine-polymer and oriented NP/polymer layers. The structure of these hierarchal assemblies was probed by X-rays as a processing parameter, N, was varied. This parameter provides a measure of the extent of *in situ* structuring by chaotic advection. We found that all assemblies are semi-crystalline at room temperature. Increasing N impacts the ratio of α to γ crystalline forms. The effects of the chaotic advection vary with the concentration of the NPs. For nanocomposites with lower NP concentrations the amount of the γ crystalline form increased with N. However, at higher NP concentrations, interfacial effects of the NP play a significant role in determining the structure, where the NPs oriented along the melt flow direction and the polymer chains oriented perpendicular to the NP surfaces.

## 1. Introduction

Controlled assembly of structured layers of polymers that contain different types of nanoparticles (NPs) offers a means to tailor multifunctional materials. NPs play an important role in modifying the properties of polymers, where incorporation of small amounts of particles significantly changes the physical characteristics of polymers [1,2,3,4,5,6]. For example, a fivefold increase in tensile strength was observed [7] when mica NPs were added to nylon (Poly Amide 6, or PA6). Significant effects were also notable on optical transparency and nonlinearity of optical response of polymers [7]. Additional effects of NPs, such as controlled permeability [8], higher thermal stability [9] and controlled electrical conductivity [10], will benefit from well-dispersed NPs. While the dispersion and orientation of the NPs affect the composite’s properties [11], effective dispersion of NPs in a polymeric matrix remains a challenge due to the tendency of NPs to aggregate [12,13]. Here we probe a new hierarchal assembly that takes advantage of inherent structuring that occurs under non-linear flow in “Smart Blending”, an extrusion technique that utilizes chaotic advection to controllably structure soft materials that are processable in a viscous liquid state [14,15,16].

Current and potential technological benefits of polymer nanocomposites lead to numerous efforts, including chemical modifications of the NPs and matrix polymers to drive assembly into desired structures [2,17]. The interaction energies between NPs and polymers, the size of NPs and their relative dimension with respect to the polymer rigid segment as well as the shape of the NP affect the assembly of the particles and the properties of the resulting nanocomposite [18,19,20,21]. Directing the assembly of NPs via chemical modifications, coupled with large-scale extrusion that can further structure the resulting composite, provide a powerful method to move processes on a lab scale to an actual technologically viable process. One such a process is a blending technique often referred to as “Smart Blending”, where chaotic advection is introduced into melts of two polymers, resulting in localization of the polymers into layers of controllable thickness and number [22,23,24,25,26]. These layers breakup due to a balance between shear and interfacial tension may lead to a wide variety of different blend morphologies. We have shown that using Smart Blending of two polymer-melts, where one contains NPs, results in hierarchical nanocomposites that consist of alternating layers of a polymer and polymer-NP layers [27].

**Figure 1:**
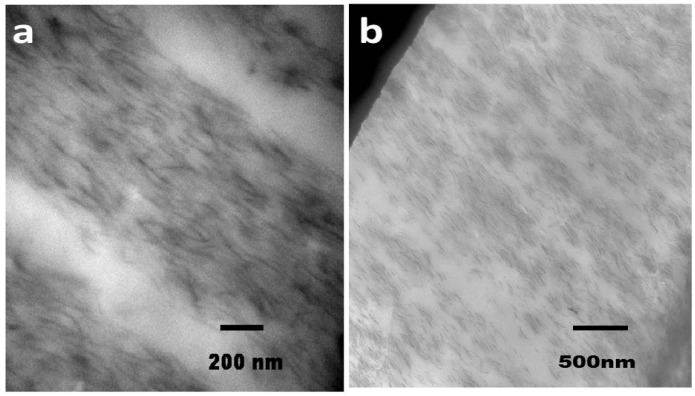
Transmission electron microscopy (TEM) image of (**a**) 2 vol % nanoparticles (NPs) montmorillonite nanocomposite formed by chaotic advection at *N =* 9 [27]; and (**b**) 5.6 vol % at *N =* 7, extruded as 150 micron thick films. Dark regions correspond to the NP rich areas and light regions correspond to the matrix polymer.

Here we probe by X-ray diffraction the assembly of molecules within layers formed by chaotic advection. Morphologies observed for of a blended PA6/PA6-montmorillonite nanocomposite, as depicted by transmission electron microscopy (TEM) are shown in Figure 1. At low and medium level of the processing parameter N, for NP of 2, a clear segregation into layers, PA6 and PA6-NPs rich layers is observed, as shown in Figure 1a [27]. With increasing N, the micron-size layers become thinner and hardly any segregation is observed between PA6 and PA6-NPs. Increasing the loading of the NP results in a composite with well oriented NPs, with lower segregation of the pristine and NP layers as shown in Figure 1b. The current study probed this non-traditional assembly of nano-particles and polymers on the nm length scale.

**Figure 2:**
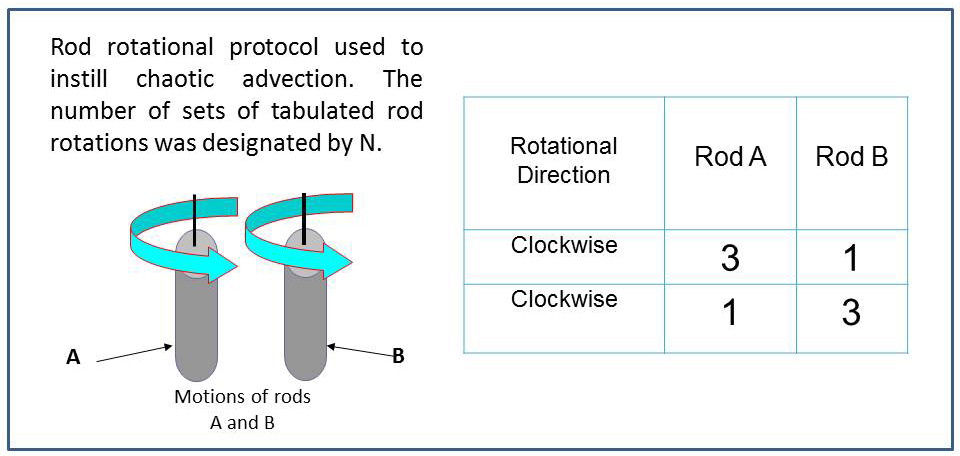
Schematic presentation of the agitator in chaotic advection and the time evolution of the slow. The agitation scheme used in the current study for *N =* 1 is presented.

The current study presents for the first time the effects of chaotic advection on the structure of hierarchal nanocomposites; *i.e.*, the effects of non-linear flows on the assembly, using X-ray diffraction. Chaotic advection is a process in which flow tracers move chaotically in response to flow fields that can be simple. Consequently, flow domains stretch and fold, resulting in exponentially fast reductions in domain size [22,23,24,25,26] forming a layered morphology. The propagation of these flows is characterized by the Lyapunov exponent by λ, where the separation of trajectories at time *t* is given by *l_t_ =* *l*_0_ *e*^λτ^ where *l*_0_ is the separation at *t* = 0. This ideal mathematical description is mitigated by finite size effects in actual blending processes. Aref [22] demonstrated that blinking vortex flows introduced by two point vortices that are alternately activated, introduce chaotic advection. This fluid mechanics study [22] led to the development of Smart Blending instruments leading to formation of new polymer blend morphologies where the extent of structuring can be regulated by selecting the duration of chaotic advection [23,24,25,26,27]. A schematic representation of the agitating rods which induce flow similar to Aref’s vortices are shown in Figure 2. The protocol for rotating the rods is also shown, where one set of motions for both rods constituted a one blending period (*N* = 1). N corresponds to a blending parameter and represents a sequence of rod-rotation.

In the experiments, several sets of such rotations were prescribed such that *N* = 5, for example, constituted five sets of rod rotations. The resulting structured melts were then extruded via a film die onto a chill roll to form films that were then studied by TEM and X-ray diffraction. Details of the smart blender are given in [25]. For polymers, quenching below their glass transition temperature retains the structures formed in the liquid phase.

Here, nanocomposites were formed in this study using two initially separate melt streams, one of nylon-6 and the other of a mixture of nylon-6/NPs. The effects of chaotic advection on the structure of the nanocomposite on a 0.1–2.5 nm length scale, where the packing of the polymer molecules is detected, were probed. PA6/montmorillonite NPs, a well-studied nanocomposite, was used as a model system. PA6 is a semi-crystalline polymer that has two major crystalline forms: α and γ. The α form consists of hydrogen bonds in between the fully extended anti parallel PA6 chains and the γ form has hydrogen bonds in between parallel pleated chains [28,29,30]. In the γ form, the plane of the amide group is roughly perpendicular to the plane of (CH_2_)_5_ groups where in the α form those planes are parallel. The α form has a monoclinic unit cell and the γ form closely resembles a hexagonal structure [31]. The α form is thermodynamically stable and the γ form is meta-stable. Previous studies have shown that adding NPs to PA6 enhances the formation of the γ form regardless of the blending technique [2,32]. Other factors that affect the type of crystalline phase formed include cooling rates [33], where enhancement of the γ crystalline form was observed when quenching by liquid N_2_ [34].

The structure at different *N* was followed by X-ray. The blend composition and the processing conditions as described in Table 1 for a given *N* and a total loading of nanoparticles. The study attempted to compare as similar as possible blends subject to experimental variability in a multistage process.

**Table 1:** Samples studied at the indicated *N* and the total loading of nanoparticles.

*N* (vol % = 0.0)	*N* (vol % = 2.0)	*N* (vol % = 2.8)	*N* (vol % = 3.5)	*N* (vol % = 5.6)
00	06	08	00	00
30	12	10	08	07
	14	12	10	09
	20	20	12	20
	30	25	16	
			20	
			22	

The results are compared with those of the pure polymer under similar conditions. The study has shown that chaotic advection not only structured the films, but also changes the ratio of the crystalline forms of the polymer depending on the concentration of the NPs, where at higher concentrations of NPs, surface effects become significant.

## 2. Results and Discussion

**Figure 3:**
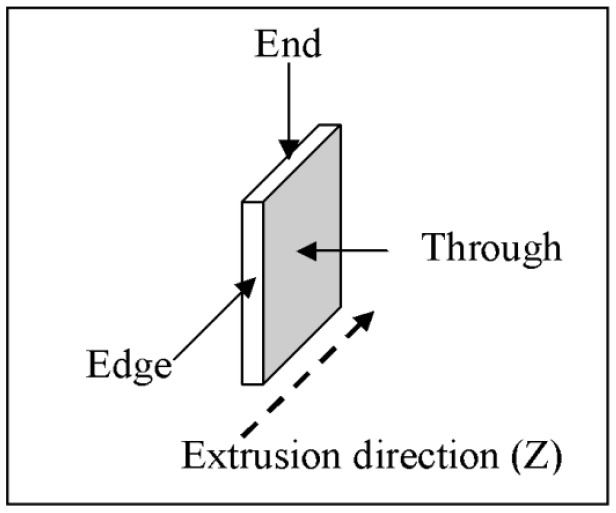
Directions of incident X-ray beams with respect to the extrusion direction. *X* and *Y* define the plane of the film and *Z* corresponds to the extrusion directions.

The structuring and assembly processes take place in the melts that are then quenched, and the resulting structure is studied by X-ray diffraction. Further details of sample preparation are given in the experimental section. The alignment of the micrometer layers as well as those of the NPs was probed along three different directions, *X*, *Y* and *Z*, with respect to the final extrusion directions as demonstrated in Figure 3.

**Figure 4:**
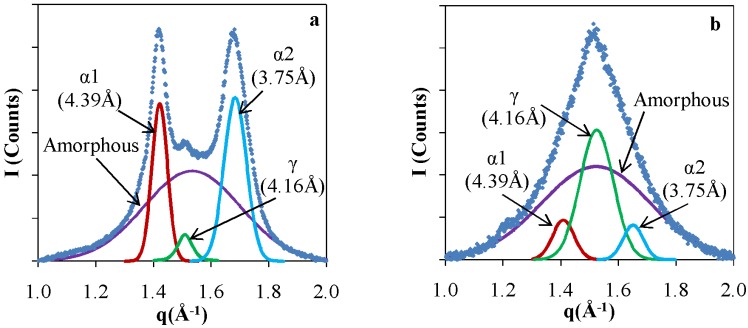
X-ray patterns and analysis of Poly Amide 6 (PA6) films (**a**) Pristine; and (**b**) Chaotically blended at *N =* 30.

In order to set the background for the NP assembly and its impact, we first probed the pristine polymer, comparing extruded PA6 in comparison with a film formed by a melt exposed to chaotic. X-ray patterns of a pristine PA6 films that were extruded without chaotic advection and otherwise identical films that were subject to *in situ* structuring by chaotic advection are shown in Figure. The pattern of the pristine polymer without chaotic advection (Figure 4a) consists of two broad peaks superimposed by a smaller one in the center. These peaks at 4.39 Å and 3.75 Å correspond to the α crystalline form and the center at 4.16 Å corresponds to the γ crystalline form. These results are consistent with previously reported data [35,36]. The α form dominates the crystalline structure of granular PA6 and exhibit two diffraction lines: 4.39 Å refers to α1 and originates from the (200) diffraction plane and 3.75 Å (α2) corresponds to the (002) plane. The peak of the γ form corresponds to the (001) diffraction plane. These diffraction lines correspond to chain-chain correlations in the crystalline domains. These values for the positions of the α and γ peaks were used to determine peak assignments in deconvolution of the nanocomposite patterns. The patterns were deconvoluted assuming a Gaussian line shape to resolve the relative amounts of α and γ as well as to determine the ratios of crystalline to amorphous fractions [33]. The results of the de-convolution are shown as solid lines in Figure 4.

The X-ray pattern of the chaotically blended PA6, shown in Figure 4b, consists predominantly of a peak that corresponds to the γ form, accompanied by two less intense α peaks. The ratios of α to γ changes from 27.4 to 0.8 and crystalline/amorphous change from 0.7 to 0.6 upon extruding the PA6 granules to make films at *N =* 30. All chaotically blended samples of the pristine PA6 had predominantly the γ crystalline form of the PA6. We attribute the formation of the meta-stable form to local shear effects that impact the alignment of the polymer chains [15].

For the nanocomposite films, extruded films from the chaotic blender consist of alternate layers of NP rich and NP free PA6 domains as shown in the TEM image in Figure 1. The X-ray data consist of average of scattering contributions from both NP-free polymer layers and layers containing the platelets. These layers are micron thick and are sufficiently thick to neglect interfacial effects arising from the PA6–PA6/NP boundaries. The effects of the NPs are extracted by comparing the results to those of PA6 exposed to chaotic advection. The differences are attributed to effects of the NPs.

**Figure 5:**
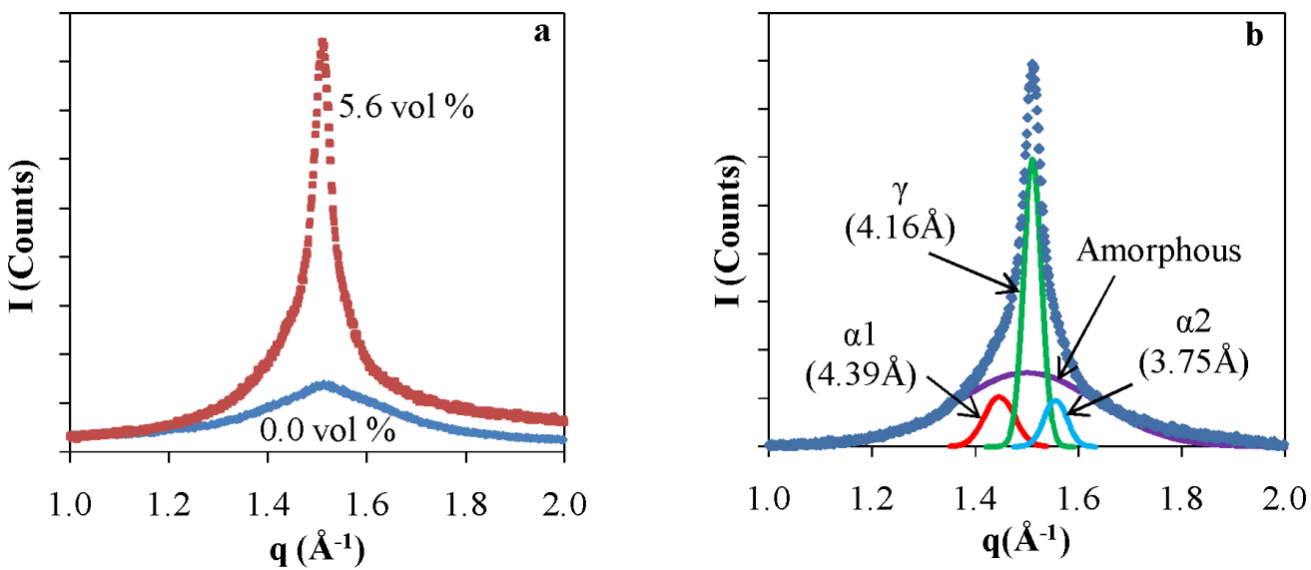
(**a**) Powder X-ray patterns of extruded PA6 with and without NPs at *N* = 0; and (**b**) Peak deconvolution of X-ray data for 5.6 vol % nanocomposite.

To form the nanocomposite films, NPs were first added and well mixed with PA6 polymer to produce a melt of desired NP concentration. Then, melts of pristine PA6 and the NP containing melt were combined in the chaotic blender and were extruded after an *in situ* structuring, where the degree of structuring is represented by the processing parameter *N*. We found that the flow impacts both the assembly of the NPs (*i.e.*, platelet orientation and confinement within PA6 layers) and the crystal structure of the polymer matrix. When NPs are imbedded in the polymer, the crystallinity of the γ form is enhanced, as shown in the X-ray patterns for extruded PA6 with and without NPs at *N =* 0 in Figure 5. For *N =* 0, the polymer melts were not subjected to chaotic advection and were simply extruded. The peak in the pure PA6 is significantly broader than that of the films containing NPs. The X-ray line width is inversely proportional to the size of the coherently scattering domains and ordering/crystalline domain size in the system. The full peak widths at half maxima δ (FWHM) are given in Table 2. Increasing NP concentration from 0 to 5.6 vol % decreases the line widths from 0.29 Å^−1^ to 0.07 Å^−1^ at N = 0. These values correspond to π/δ, the size of the coherently scattering domains, of ~20 Å to ~90 Å. With increasing NP concentrations the interfacial area between the NPs and the polymers increases, enhancing the correlation between the chains.

**Table 2:** X-ray peak widths for nanocomposite films as a function of *N*, the processing parameter and NP concentration in terms of volume percentage marked by vol %.

NP vol %	*N*	FWHM (δ) (Å^−1^) (±0.001)	NP vol %	*N*	FWHM (δ) (Å^−1^) (±0.001)	NP vol %	*N*	FWHM (δ) (Å^−1^) (±0.001)
0.0	0	0.29	2.0	12	0.07	5.6	07	0.06
4.0	0	0.14	2.0	14	0.06	5.6	09	0.09
5.6	0	0.07	2.0	20	0.07	5.6	20	0.08
			2.0	30	0.07			

In contrast to the significant effects of *N* shown on the micron length scale, *N*, which was varied from 0 to 30, hardly affected the domain sizes over which the polymer chains scatter coherently.

**Figure 6:**
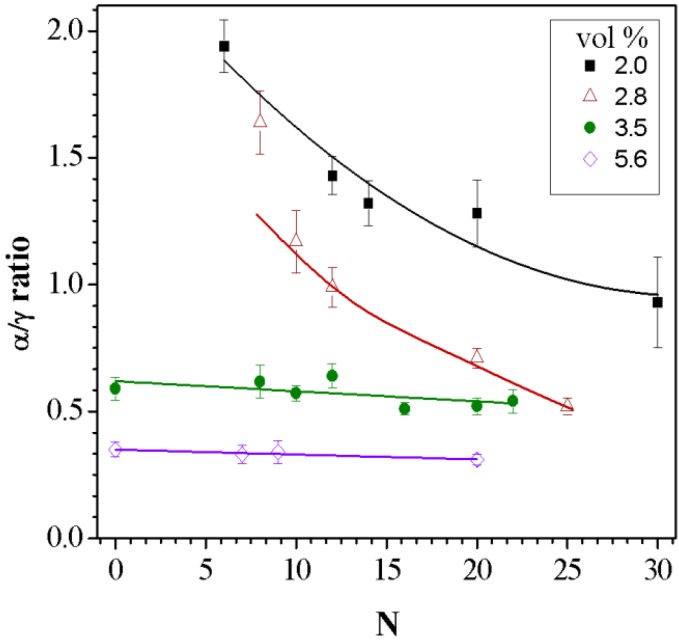
Variation of α/γ crystalline ratios with the processing parameter *N* for samples at the indicated NP compositions. The solid lines are drawn as a guideline for the eye.

The fractions of the α and γ crystalline are presented in Figure 6 as the ratio of the two crystalline forms (α/γ) as a function of *N* for different NP concentrations. At low NP concentrations, 2.0 and 2.8 vol %, the ratio of α/γ decreases with increasing *N*, where at higher concentrations this ratio remains constant. In low NP concentrations, blending at higher *N* values results in enhancing the γ crystalline form. This trend at low NP concentration is similar to that observed in pure PA6 films where α/γ ratio decreases from 1.8 to 0.8 as *N* goes from 0 to 30. Enhancing the NP concentration increases the surface area between the polymer and the NP. A large surface area results in increase cohesiveness of the polymer and NP affecting the polymer orientation. 

Though increasing the NP content affects the melting temperature, *T*_m_, of the polymer and the overall melt viscosity, the blending temperatures are sufficiently above *T*_m_ that *in situ* structuring occurs nearly independently for the NP concentrations studied.

**Figure 7:**
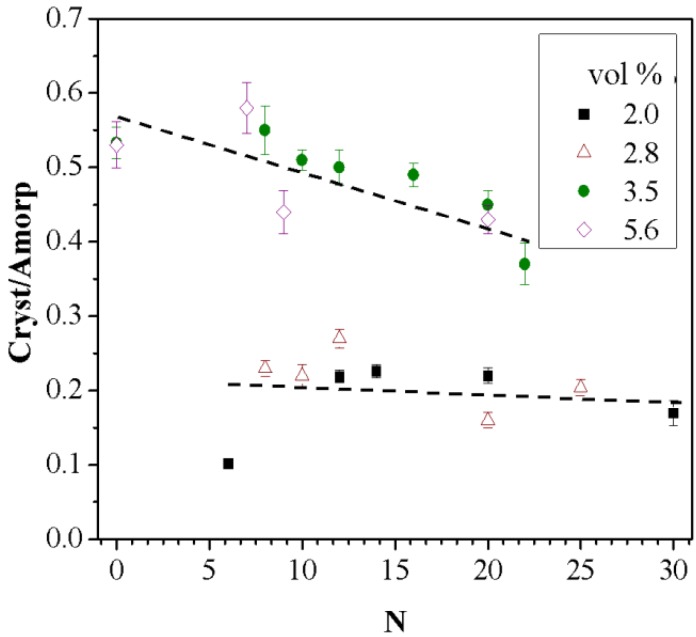
Total crystalline/amorphous ratio variation with respect to the processing parameter *N* at the indicated NP compositions. Lines are drawn as a visual guide.

The results show that while chaotic advection dominates at low NP concentrations, surface interactions of the NPs with the polymer chains control the system at higher NP concentrations. The overall degree of crystallinity is affected by both *N* and the concentration of NPs. The total crystalline fraction present in the nanocomposite blends, as extracted from the peak deconvolution, is shown in Figure 7. This fraction has contributions from both α and γ crystalline forms. Nanocomposites with 2.0 vol % and 2.8 vol % NPs are of lower crystallinity compared to higher NP concentrations. This is attributed to enhanced fraction of confined polymer chains within NPs, which facilitates the formation of more crystalline domains. However, no significant effects were observed as a function of *N*.

**Figure 8:**
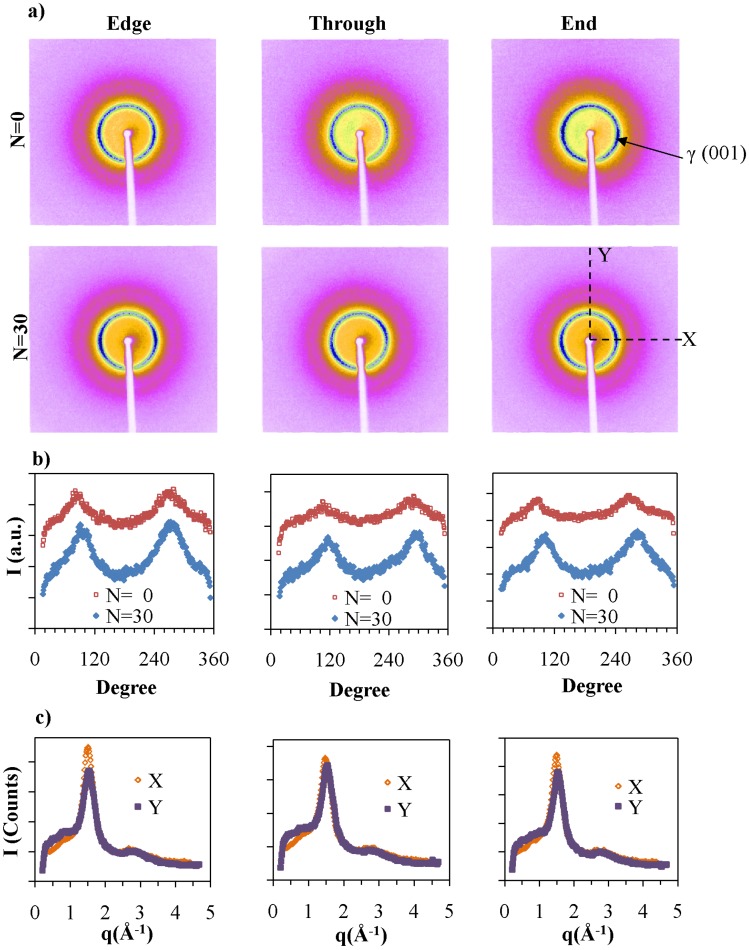
(**a**) Two-dimensional X-ray pattern of PA6 films at *N =* 0 and *N =* 30 from edge, through and end directions. The white regions in the middle of the images correspond to the shadow of the beam stopper; (**b**) The intensity along the γ crystalline ring marked in γ for the different directions; and (**c**) The cross sections of the *N =* 30 along the *X* and *Y* directions.

Additional experiments were carried out using a two-dimensional detector to follow effects of *N* and the NP concentration on the orientation of the components. The X-ray patterns were measured in three directions with respect to the film plane and the direction of extrusion. The patterns corresponding to PA6 at *N =* 0 and *N =* 30 are shown in Figure 8. The intensity of the lines is affected by the amount of material in the X-ray beam. The first intense diffraction line from the center corresponds to the γ (001) diffraction of PA6, which is observed in all three directions. Two less intense arcs are observed corresponding to γ (001) diffraction in *Edge* and *End* patterns along the equator. In PA6 extruded different ways, no orientation is observed, and the intensity of this line is increased with *N*. We attribute the alignment observed to the effects of the chaotic advection, where the chains lie in the direction of the flow and the (001) plane lies perpendicular to the long axis of the polymer chain. The intensity of the γ (001) diffraction was integrated along the diffraction ring to obtain the radial distribution of the γ form. The results are shown in Figure 8b, where we observed the two peaks, which correspond to the intense arcs in the equatorial direction. The cross sections of *N =* 30 patterns in the *X* and *Y* directions further confirms the slight intensity difference of γ (001) along equatorial and meridial directions as indicated in Figure 8c. For pure PA6 there is no specific orientation as observed in injected molded samples [37,38,39]. Therefore, orientation of γ form observed in pure PA6 is due to the alignment of polymer chains in chaotic flow fields.

NPs enhance the packing and orientation of the polymer chains. The 2-D patterns for 5.6 vol % NP nanocomposites are presented in Figure 8a. The most center diffraction, at ~18 Å, corresponds to the inter-platelet distance between NPs. This diffraction spot is clearly observed in the *Edge* and *End* views and not in the *Through* direction. As indicated in Figure 8c, the diffraction of intercalated NPs occurs only along the equator for *Edge* and *End* directions and not from the meridial direction. Therefore the long axis of the particles orient along the extrusion direction as was previously shown by TEM. *N* hardly affects the platelets orientation. These results are consistent with previously reported studies of PA6/NP nanocomposites that were prepared by injection molding [2].

**Figure 9:**
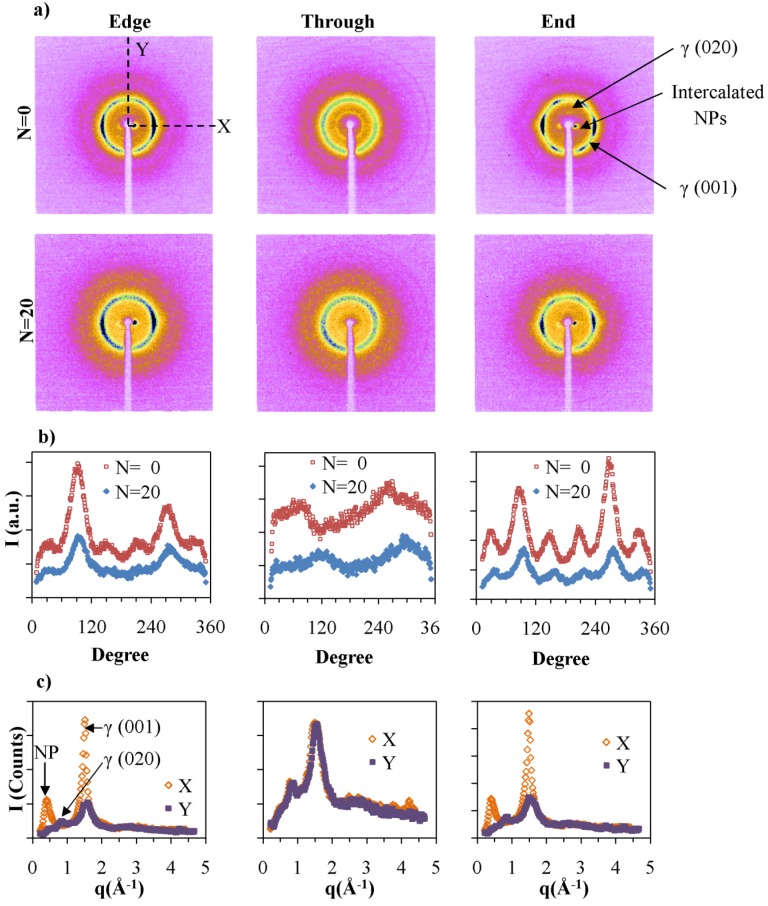
(**a**) Two dimensional X-ray patterns of PA6 with 5.6 vol % NP nanocomposite films at *N =* 0 and *N =* 20 from edge, through and end directions; (**b**) The intensity along the γ crystalline ring marked in γ for the different directions; and (**c**) The cross sections of the *N =* 0 along *X* and *Y* directions.

In the *Through* direction, the γ form diffraction line of PA6 is isotropic. However, in *Edge* and *End* directions, a six-fold symmetry in (001) is observed. As shown in Figure 9c, the intensities of the arcs along the equator are higher than the ones closer to the meridian. The radial distributions of γ form of PA6 for 5.6 vol % NP at *N =* 0 and *N =* 20 presented in Figure 9b, show the six-fold symmetry and relative intensities of arcs. The data are consistent with both hexagonal and monoclinic structures. The monoclinic unit cell of γ form was observed when PA6 is treated with iodine to induce the γ crystalline form [31]. The melt spun PA6 have pseudo-hexagonal symmetry [39], whereas injection molded PA6 nanocomposites exhibit a six-fold symmetry due to the presence of pseudo-orthorhombic lattice. In the current study we have observed a limited number of diffraction peaks, which do not allow us to distinguish crystalline packing that corresponds to the six-fold symmetry.

**Figure 10:**
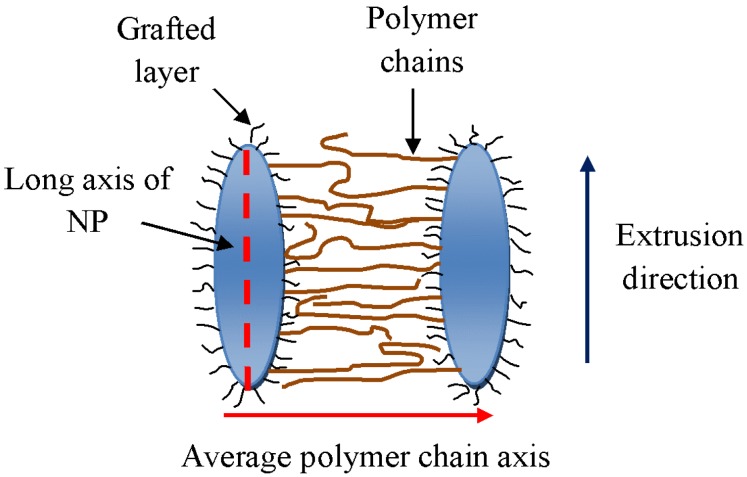
A schematic representation of the relative orientations of NPs and PA6 chains in nanocomposites.

The γ (001) diffraction is in a plane perpendicular to the polymer chain axis. Since orientation of PA6 can only be observed in the *Edge* and *End* configurations, polymer chain axis lies on average perpendicular to *Edge* and *End* scanning directions. Therefore, these polymer chains are oriented perpendicular to the flow direction as well as to the surface of NPs as shown in Figure 10. Generally, polymer chains arrange parallel to the surfaces of the internal layers containing them. Such layers were themselves formed by shear acting predominantly in the Smart Blender in the direction perpendicular to the extrusion direction. This effect is consistent with studies by Kumar and co-workers [19] that observed interpenetration of the polymer matrix into the grafted layer on the NP.

The (020) diffraction is observed at 8.14 Å along the meridian of the *Edge* and *End* patterns, which further confirms the perpendicular orientation of polymer chains with respect to NP surface. The diffraction spot for the (020) plane is observed in samples with lower *N* values. With increasing *N*, the (020) diffraction is no longer visible.

## 3. Experimental Section

Master-batches, a mixture of the polymer with NPs of different NP percentages, were prepared from PA6 (Capron B135QP, BASF Corporation, Mount Olive, NJ, USA) and NP (Closite 30B, Southern Clay Products, Gonzales, TX, USA) using a twin-screw extruder [39]. Corresponding glass transition (*T*_g_) and melting temperatures (*T*_m_) of the master-batches were measured using differential scanning calorimeter (DSC) and the corresponding values are given in Table 3.

**Table 3:** Glass transition (*T*_g_) and melting temperatures (*T*_m_) of master batches with different NP volume fractions (vol %).

vol %	*T*_g_(°C) (±0.2)	*T*_m_(°C) (±0.2)
0 0	45.2	220.1
0 5	43.4	218.8
10	46.8	220.7

DSC measurements were performed on a Mettler Toledo 910e model (Greifensee, Switzerland) with flow rates of 10 °C/min. Samples were sealed in aluminum pans to avoid contact with air during the measurements. Nanocomposite films with NP volume fractions (vol %) of 2.0%, 2.8%, 3.5%, and 5.6% were obtained by introducing the matrix PA6 polymer and master-batches in equal proportions to the smart blender to obtain extruded films of about 150 micron thickness. The volume is reported as % of the total PA-6 in the system. Films were produced with differing extents of chaotic advection controlled through specification of *N*; a parameter that specifies the motion of rods that blend the polymers [22]. A list of samples measured and their volume fractions are given in Table 3.

An X-ray powder Sintag diffractometer, with Cu K_α_ radiation and wavelength of λ = 1.54 Å, was used to obtain powder X-ray patterns. All the samples were cut to a 1 cm^2^ films. The scattering intensities were normalized to a unit thickness. Further studies were carried out on a Rigaku single crystal instrument with Mo *K*_α_ radiation λ = 0.71 Å, equipped with a two-dimensional detector. The patterns were recorded along three different directions with respect to extrusion direction as shown in Figure 3. Degrees of crystallinity and α/γ ratios were calculated from X-ray data with the PeakFit 4.2 program, which de-convolutes overlapped peaks and integrate the peak areas. Two-dimensional X-ray images were analyzed using MATLAB incorporating a home built intergraded routine to obtain radial distributions and intensity profiles along different directions.

## 4. Conclusions

The assembly of a hierarchical PA6/montmorillonite NP composite was investigated at different NP concentrations as the extent of *in situ* structuring by chaotic advection was varied. The segregation into two defined layers, a pristine polymer free of NPs and a NP-polymer compounded layer, is a direct result of recursive stretching and folding that characterizes chaotic motion. We found that the internal structure of the layers is a result of balance between forces induced by the flow and chaotic advection as well as interfacial forces excreted by the surface of the NPs that are present throughout the assembly process. These forces impact both the crystalline phases and the degree of orientation of the nanoparticles. At low loading of NPs, the α crystalline form transform into the γ form as layers become thinner, whereas at higher loadings no changes were observed in response to the extent of chaotic advection. At higher loadings, crystalline morphology is determined largely by the smaller inter-platelet distances. In nanocomposites, polymer chains align perpendicular to interface of the NPs. This study has demonstrated that interfacial interactions in nanocomposites could be sufficiently strong to impact the nm structure features, whereas chaotic flows impact the micron level structuring. Further studies including varying the dimensions of the NPs correlated with the degree of structuring are on the way to quantify the impact of chaotic advection on structuring of polymeric nanocomposites.
